# Curcumin Targeting Non-Coding RNAs in Colorectal Cancer: Therapeutic and Biomarker Implications

**DOI:** 10.3390/biom12101339

**Published:** 2022-09-21

**Authors:** Jiaying Li, Rundong Chai, Yinxiao Chen, Shuwu Zhao, Yuhong Bian, Xiangling Wang

**Affiliations:** College of Integrative Medicine, Tianjin University of Traditional Chinese Medicine, No.10 Poyanghu Road, JingHai District, Tianjin 301617, China

**Keywords:** curcumin, colorectal cancer, non-coding RNAs, epigenetic regulation, anti-tumor

## Abstract

Colorectal cancer is one of the most common gastrointestinal malignancies, with high incidence rates, a low rate of early diagnosis, and complex pathogenesis. In recent years, there has been progress made in its diagnosis and treatment methods, but tumor malignant proliferation and metastasis after treatment still seriously affect the survival and prognosis of patients. Therefore, it is an extremely urgent task of current medicine to find new anti-tumor drugs with high efficiency and safety and low toxicity. Curcumin has shown potent anti-tumor and anti-inflammatory effects and is considered a hot spot in the research and development of anti-tumor drugs due to its advantages of precise efficacy, lower toxic side effects, and less drug resistance. Recent studies have revealed that curcumin has anti-tumor effects exerted on the epigenetic regulation of tumor-promoting/tumor-suppressing gene expression through the alteration of expression levels of non-coding RNAs (e.g., lncRNAs, miRNAs, and circRNAs). Herein, we summarize the interaction between curcumin and non-coding RNAs on the occurrence and development of colorectal cancer. The information complied in this review will serve as a scientific and reliable basis and viewpoint for the clinical application of non-coding RNAs in colorectal cancer.

## 1. Introduction

Colorectal cancer (CRC) ranks third in the incidence of malignant tumors and is the second leading cause of cancer-related death worldwide [1,2]. According to the global cancer statistics issued by International Agency for Research on Cancer (IARC), 1.9 million new cases (third highest incidence) and 935,000 colorectal cancer deaths (second highest mortality) were estimated to have occurred worldwide in 2020 [3]. The global burden of colorectal cancer is expected to be more than 2.2 million new cases a year and 1.1 million deaths in 2030 [4]. In China, the incidence and mortality of colorectal cancer have also increased due to variations in diet and the population age structure [5,6]. Due to the lack of early specific warning signs of colorectal cancer, most patients are in phases III and IV at the first visit and might lose the opportunity to receive effective standard treatment, resulting in a 5-year survival rate of 40% [7,8]. Therefore, it is urgent to find new therapeutic methods and develop effective biomarkers for the early diagnosis, treatment, and prognosis assessment of colorectal cancer to improve the survival status. Some studies have demonstrated that epigenetic mechanisms play a key role in cancer progression, particularly non-coding RNAs (ncRNAs) [9]. Indeed, many studies have demonstrated that the development pathogenesis of colorectal cancer is highly influenced by ncRNAs [10], the abnormal expression of oncogenic and tumor-suppressor molecules, and the abnormal activation of various cell signaling pathways [11,12]. In recent years, some important natural compounds, such as phenolics, terpenoids, and meroterpenoids, have been confirmed to have anticancer effects by regulating the expression and function of ncRNAs [13]. Particularly, naturally derived polyphenols have been safely used for many years and have shown real potential for therapeutic effects in most cancers through the regulation of miRNAs and lncRNAs [14,15,16]. The initial epigenetic changes associated with cancer may be regulated by many polyphenols [17,18,19], such as curcumin [20,21], resveratrol [22,23], and so on. Among them, curcumin, with the advantages of less toxicity and less side effects, has been clarified to be an effective compound in the treatment of colorectal cancer. Therefore, summarizing the scientific progress of curcumin targeting ncRNAs in colorectal cancer and understanding the inducement and molecular regulatory mechanisms of colorectal cancer is essential in finding key targets for clinical treatment and will also provide theoretical guidance for basic research, clinical drug selection, and gene therapy.

### 1.1. Curcumin and Cancer

Curcumin, also known as diferuloylmethane, is a natural active ingredient extracted from the rhizome of *Curcuma longa* [24,25]. Curcumin is an orange–yellow crystalline powder [26,27] with minimal toxicity [28] and a kind of fat-soluble polyphenol, with the main chain containing unsaturated aliphatic and aromatic groups [29]. It has been approved as a food additive by the World Health Organization and the US Food and Drug Administration [30] and has been listed as a third-generation cancer chemoprevention drug by the National Cancer Institute of the United States due to its safety, non-toxicity, and lack of adverse effects [31]. To date, curcumin products that are water-soluble and oil-soluble have been developed in several forms in China [32], including capsules, tablets, ointments, energy drinks, soaps, and cosmetics; these are widely used in many different areas, such as health care, food, medicine and cosmetics [33].

Curcumin has a non-toxic chemical composition; almost all types of tumor markers can be regulated by it to exert an anti-tumor effect; undoubtedly, this provides a strong theoretical basis for cancer treatment [34,35,36,37]. Studies have shown that curcumin could exert an anti-tumor effect both in vitro and in vivo through different mechanisms, including inhibiting the invasion, metastasis, and proliferation of tumor cells, inducing tumor cell apoptosis and autophagy, and resisting chemotherapy resistance [38,39,40]. In addition, abnormal epigenetic modification is closely related to tumorigenesis and runs through all stages of tumors. Particularly, the precise regulation of ncRNAs based on epigenetic regulation in various biological processes plays a vital role in the occurrence and development of tumors. Single ncRNA can modulate the expression of multiple downstream target genes and associated pathways, which provides a theoretical basis for the development of cancer therapeutic drugs. Recent studies have shown that curcumin is widely used as an anti-tumor agent because it regulates ncRNAs based on an epigenetic regulation mechanism [41,42,43]; there are benefits to using curcumin in the treatment of colorectal cancer.

### 1.2. Non-Coding RNAs and Cancer

**Figure 1:**
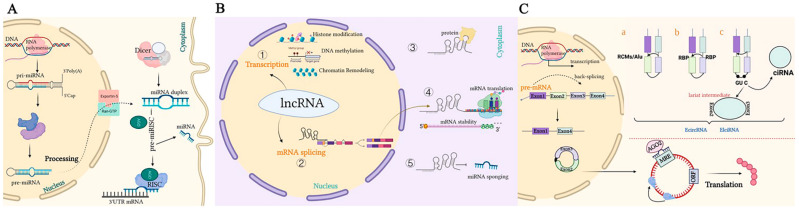
Representation of the biogenesis and mode of action of ncRNAs. (**A**). A pri-miRNA with a double-stranded stem-loop is formed after transcription, which is cleaved by Drosha and DGCR8 with a hairpin-based secondary structure; two nucleotides overhang at its 3′ end. Then, pre-miRNAs are exported to the cytoplasm by exportin-5/Ran-GTP. Here, pre-miRNA forms a mature double-stranded miRNA duplex digested by Dicer. The miRISC complex formed by the guide strand miRNA and Ago protein represses target mRNAs by base-pairing at 3′UTR, which prevents translation and selectively silences gene expression. (**B**). Mechanisms underlying long non-coding RNA (lncRNA)-mediated regulation of gene expression. ① Transcription regulation by lncRNAs. ② lncRNAs are engaged in the processing and maturation of mRNAs ③ lncRNAs interact with proteins. ④ lncRNAs interact with RNAs. ⑤ lncRNAs can competitively bind to miRNAs by acting as ceRNAs, thereby blocking the inhibition of the target gene. (**C**). Biogenesis of circRNAs. a. The back-splicing circularization requires the help of complementary sequences (ALU repeats and RCMs). b. RBP-mediated circularization. c. Lariat-driven circularization. circRNA can serve as an miRNA sponge, which inhibits miRNAs in order to regulate the expression of target genes or interact with proteins.

The human transcriptome contains mostly non-coding RNAs (ncRNAs) that exist in different cell types (including normal cells and tumor cells) [44,45] and are transcribed from DNA but not translated into proteins [46]. These are functional RNA molecules that participate in the various stages of gene expression regulation, which include microRNAs (miRNAs) [47], long non-coding RNAs (lncRNAs) [48], and circular RNAs (circRNAs) [49]. Figure 1 shows the biogenesis and mode of action of miRNAs, lncRNAs, and circRNAs.

miRNAs, about 18 to 25 nucleotides in size, are small ncRNA molecules with regulatory functions [50]. In most mammals, miRNAs can destroy the stability or interfere translation of mRNAs through complete or incomplete base pairing with the 3′ untranslated regions (3′UTRs) of their target mRNAs, which can precisely regulate their target gene expression process [51]. miRNAs can form a complex regulation network by regulating related signaling pathways that have an important implication in tumor development and chemotherapy resistance [52]. Meanwhile, the precise regulation of miRNAs also plays a crucial role in the process of autophagy [53]. In the early stage of tumorigenesis, autophagy can act as a tumor suppressor, which restores homeostasis and eliminates cancerous cell components. However, in the advanced stage of tumors, autophagy may promote tumor growth and metastasis, which may produce therapeutic resistance [54]. Compared with normal tissues, the abnormal expression of miRNAs in cancer tissues is mainly manifested as the downregulation or deletion of miRNAs with tumor-suppressor functions and the overexpression of miRNAs with oncogenic functions [55]. Examples of well-characterized tumor-suppressor miRNAs include miR-34a, miR-145, and the let-7 family; well-established oncogenic miRNAs include miR-21 and miR-155 [56,57,58]. Interestingly, several miRNAs appear to possess dual functionality, acting as both a tumor suppressor and an oncogene. For instance, although miR-200c inhibits epithelial-to-mesenchymal transition (EMT) and blocks the initiation of cancer metastasis, it is also frequently overexpressed in late-stage cancers and is involved in promoting distant metastasis [59,60]. Therefore, increasing evidence suggests that the dysregulation of miRNAs could alter the physiological processes of cells and regulate a wide variety of biological processes, such as tumor growth, proliferation, apoptosis, chemoresistance, migration, and invasion [61,62,63,64,65].

LncRNAs, more than 200 nucleotides in length, are a group of transcription products regulated by RNA polymerase II [66]. LncRNAs can be classified according to their relevance to protein-coding genes. Additionally, there are certain subgroups, such as overlapping lncRNAs, which contain a protein-coding gene in their intron; divergent lncRNAs that are transcribed from the opposite direction of an adjacent protein-coding gene; intronic lncRNAs, whose total nucleotide sequence fits with the intron of a protein-coding gene; intergenic lncRNAs, whose nucleotide sequence is associated with two genes; and lncRNAs in antisense transcript form, which reside between exons of other transcripts at the antisense strand [67]. Based on their important roles in the regulation of gene expression, they participate in several physiologic and pathologic processes, including the development of cancer [68]. Even though many lncRNA-coding genes have histone modification signatures distinct from mRNA-coding genes, these non-coding RNAs are also produced and transcribed by RNA polymerase II, such as protein-coding RNAs (mRNAs). After being transcribed, lncRNAs are processed through 5′ end-capping, intron splicing, 3′ end-polyadenylation, and intracellular transportation, similar to mRNAs [69]. The available evidence indicates that lncRNAs could interact with macromolecules such as DNA, RNA, and proteins to mediate the regulation of gene transcription and post-transcriptional processes such as endogenous gene expression, mRNA splicing and modification, and protein translation, which influence tumorigenesis and development [70,71,72]. Additionally, lncRNAs could be used as competitive endogenous RNAs (ceRNAs) to absorb miRNAs and affect both target mRNA translation and protein synthesis [73]. As mentioned above, lncRNAs may act as tumor-promoting or tumor-suppressing genes and potential diagnostic and prognostic markers of the progression and chemoresistance of colorectal cancer.

circRNAs, a new type of ncRNAs [74,75], are formed in a covalently closed continuous loop through the ligation of the 5′ and 3′ ends of linear RNAs [76]. This structural feature makes circRNAs resistant to digestion by ribonucleases (such as exonuclease R (RNase R) and exonuclease), with higher stability and longer half-life than linear mRNAs. CircRNAs are largely generated from exonic or intronic sequences, and reverse complementary sequences or RNA-binding proteins (RBPs) are necessary for circRNA biogenesis [77,78]. In addition, studies have also found that circRNAs are mostly composed of exons, and a few are formed by intron circularization, which is highly conserved in evolution among species [79] and has timing specificity in certain tissue cell sources and different developmental stages [80]. Recent studies have revealed that circRNAs have a variety of functions, including acting as a miRNA sponge (ceRNA), interacting with proteins, regulating alternative splicing, and transcribing parental genes [81,82,83,84]. circRNAs have been found to act as miRNA sponges and are involved in the occurrence and development of a variety of cancers through the circRNA–miRNA–mRNA axis. circRNAs specifically absorb miRNAs to regulate their expression and indirectly control the expression of proteins. After miRNAs and lncRNAs, circRNAs have become a new hotspot in the field of ncRNAs in recent years [85]. In addition, the differential expression of circRNAs in tumors is thought to play an important role in the malignant behavior of various tumors, such as cell cycle, cell proliferation, apoptosis, invasion, and chemotherapy resistance; this has given us a novel recognition that circRNAs could be used as new tumor biomarkers and therapeutic targets [86].

In addition, a large number of studies have shown that the dysregulation of ncRNAs contributes to the development of cancer drug resistance by modulating the expression of specific target genes involved in cellular apoptosis, autophagy, drug efflux, epithelial-to-mesenchymal transition (EMT), and the cell cycle [87,88]. Notably, ncRNAs are well known as crucial regulators of autophagy through the regulation of ATGs and autophagy-associated signaling pathways, including the phosphatidylinositol 3-kinase (PI3K)/protein kinase B (AKT)/mammalian target of rapamycin (mTOR) signaling pathway, which ultimately mediates chemoresistance and radioresistance [89,90]. This provides an important breakthrough for the role of ncRNAs in the treatment resistance of malignant tumors and also highlights the interaction between molecules.

Overall, there is increasing evidence that the abnormal expression of ncRNAs is involved in the pathology of various tumors. The miRNAs, lncRNAs, and circRNAs involved in colorectal cancer could exhibit either tumor-suppressing or -promoting effects, which may be useful in the diagnosis and targeted therapeutics.

## 2. Curcumin and Colorectal Cancer Therapy Based on Non-Coding RNAs’ Epigenetic Regulation

### 2.1. Curcumin against Colorectal Cancer Mediated by miRNAs

Many studies have demonstrated that the occurrence, development, treatment, and prognosis of colorectal cancer are all involved with miRNAs in varying degrees [91,92]. miRNAs can regulate the expression of their target oncogenes or cancer suppressor genes to affect the various biological processes of cancer, including cell proliferation, apoptosis, cell cycle, and metastases, in the occurrence and development of malignant tumors [93]. Some studies have indicated that curcumin could exert anti-colorectal cancer effects by targeting differentially expressed miRNAs [94,95]. The miRNAs regulated by curcumin in colorectal cancer are summarized in Table 1.

miR-21 is considered one of the important tumor-promoting genes, and its abnormally high expression is closely related to the proliferation and invasion of different tumor cells. For example, curcumin inhibits the invasion and metastasis of colon cancer cells (Rko and HCT116) by specifically targeting and downregulating the level of oncogenic miR-21 [96]. Moreover, some studies have revealed that curcumin blocks the binding of activator protein-1 (AP-1) in the promoter region of miR-21, promoting the expression of tumor suppressor programmed cell death protein 4 (PDCD4), which is suppressed by miR-21 in phorbol-12-myristate-13-acetate (PMA) stimulated colorectal cancer [97,110]. This, to a certain extent, explains the targeted inhibition and regulation of curcumin on miR-21. Interestingly, Shao et al. found that after treating human colorectal cancer cell HCT116 with curcumin, the level of apoptosis and autophagy were dose-dependent on curcumin and decreased the expression of miR-21-3p and miR-21-5p, which increased the expression of ATG10 and APAF1 and promoted autophagy and apoptosis in HCT116 cells [98].

Conversely, miR-491, as a tumor suppressor, is aberrant underexpressed in colorectal cancer cells. It not only inhibits the proliferation of colorectal cancer cells but also prevents the invasion of these malignant cells [111,112]. It was observed that the expression of miR-491 was increased after exposing colorectal cancer cells to curcumin, which could downregulate the expression of PEG10 subsequently by binding to its 3′-UTR [101]. This pathway sensitizes colorectal cancer cells to apoptosis and inhibits proliferation. However, curcumin induces the downregulation of miR-27a and upregulation of miR-34a, resulting in cell cycle arrest and apoptosis in colorectal cancer cells. Previous studies have shown that combined with acetyl-11-keto-β-boswellic acid (AKBA), curcumin could effectively inhibit mouse xenograft tumor growth by further downregulating the expression of miR-27a and the overexpression of miR-34a [100].

Furthermore, epithelial–mesenchymal transition (EMT) is an initial stage of the metastasis process and is closely related to tumor chemotherapy resistance in cancer. EMT tumor cells gain the ability of apoptosis resistance and maybe result in tumor resistance to radiotherapy and chemotherapy, which seriously affects the prognosis of cancer patients [113]. Notably, miRNAs play critical roles in the regulation of EMT [114,115]. There is an experiment that described that curcumin increased the expression of tumor-suppressor miRNAs (such as miR-34a, miR-200c, miR-141, miR-429, and miR-101) by enhancing the chemosensitivity of colorectal cancer cells to 5-fluorouracil (5-FU); thereby, the prevention of EMT can inhibit the metastasis of colorectal cancer cells [102]. Kara et al. found that abnormally high expressions of miR-130a are associated with chemotherapy resistance and lead to poor clinical chemotherapy outcomes in patients with colorectal cancer [116]. Curcumin mediates the downregulation of miR-130a and can promote the activation of the Wnt/β-Catenin signaling pathway in colorectal cancer; this enhances chemosensitivity and inhibits the proliferation of mouse colorectal cancer cells [99]. Additionally, Han et al. reported that curcumin could reverse the resistance of colorectal cancer cells to oxaliplatin (L-OHP) chemotherapy by affecting ERCC1, the expression of which is mediated by miR-409-3p [103]. Moreover, a recent study proved that curcumin could overcome cisplatin resistance by inhibiting miR-137-mediated glutamine metabolism [108]. Therefore, curcumin may become an ideal drug candidate for anti-chemotherapy resistance in colorectal cancer.

In conclusion, curcumin precisely regulates the physiological functions of its target genes by regulating the expression of miRNAs, affects various stages of colorectal cancer, including occurrence, development, treatment, and prognosis, and is of great significance when exploring new therapies for the treatment of colorectal cancer.

### 2.2. Curcumin against Colorectal Cancer Mediated by LncRNAs

LncRNAs, as effective marker molecules, have been used in the diagnosis and prognosis of many cancers. The abnormal expression of some lncRNAs is involved in various processes, such as regulating the growth and metastasis of tumor cells [117]. Curcumin could significantly change the proliferation, migration, and invasion of colorectal cancer cells by regulating lncRNAs [118]. Furthermore, the mechanism of lncRNAs that interact with curcumin to moderate signal transduction needs to be further explored. Therefore, in this section, we will discuss the intervention effect between curcumin and lncRNAs in colorectal cancer and its anti-colorectal cancer mechanism.

A recent study found that the upregulation of lnc NBR2 (neighbor 2 of BRCA1 lncRNA) could inactivate the mTOR signaling pathway while opening another positive feedback pathway to enhance AMPK potentiation by energy metabolism stress so as to inhibit the proliferation of colorectal cancer cells. Curcumin could significantly increase the expression of lnc NBR2 in this process. The inhibitory effect of curcumin on colorectal cancer cells disappeared when lnc NBR2 was knocked down. These data demonstrate that the anti-tumor mechanism of curcumin in colorectal cancer is dependent on the activation of lnc NBR2 and AMPK signaling [119].

LncRNA KCNQ1 opposite strand/antisense transcript 1 (KCNQ1OT1) is located at the KCNQ1 cluster on human chromosome 11p15.5 and is highly associated with the development of cancer [120]. It has been observed that KCNQ1OT1 is highly expressed in colorectal cancer cells, and its potential mechanism could act as a sponge for miR-497 (a Bcl-2 inhibitory miRNA) [121]. Zheng et al. have reported that curcumin significantly inhibits the proliferation and promotes the apoptosis of colorectal cancer cells in a dose-dependent manner based on the lncKCNQ1OT1–miR-497–Bcl-2 axis. In contrast, overexpression of KCNQ1OT1 could reverse the anti-proliferative function of curcumin and increase Bcl-2 levels to promote cisplatin resistance in colorectal cancer cells [122].

Cellular senescence is a proliferative arrest bioprocess by potentially cancer-promoting factors, and it can limit the outgrowth of pre-cancerous cells [123]. Lnc PANDAR (promoter of CDKN1A antisense DNA damage-activated RNA) is located at chromosome 6p21.2. Notably, its upregulation inhibited the activation of pro-apoptotic genes (NOXA, PUMA, and FAS) that could induce cellular senescence. The depletion of lnc PANDAR could delay senescence through the stimulated gene expression of CCNB1, CDK1, and CDC25C and increase apoptosis in colorectal cancer cells [124,125]. Similarly, a recent study proved that simply silencing lnc PANDAR expression could not change the proliferation, apoptosis, and senescence processes of colorectal cancer cells, whereas low-does curcumin combined with the silencing of lnc PANDAR could promote apoptosis and delay cellular senescence in colorectal cancer cells. It was indicated that this effect may be mediated by the induction of PUMA (p53 upregulated modulator of apoptosis) expression, which has a significant reference value for the treatment of colorectal cancer [126].

In addition, an in vitro study revealed that lnc MALAT1 (metastasis-associated lung adenocarcinoma transcript 1 lncRNA) has an abnormally high level of expression in colorectal cancer tissues. Curcumin combined with si-MALAT1 could significantly reduce its expression and inhibit the cell activity, migration, and invasion of SW480 (colorectal cancer cell) [127]. Through polymeric hybrid nanoparticles (CSNPs), combining curcumin with siCCAT1 (Lnc CCAT1 small interfering RNA) could effectively inhibit the proliferation and migration of HT-29 cells (colorectal cancer cells) and induce a high apoptosis rate in a synergistic manner [128]. Hence, nanoparticles packaged with overexpressed/silenced Lnc RNAs and collaboratively transported with curcumin may provide a better and more promising strategy for colorectal cancer treatment. As a consequence, the above-mentioned results have gradually provided new evidence for the basic research of lncRNAs combined with curcumin for the treatment of colorectal cancer. The data are shown in Table 2.

### 2.3. Curcumin and Anti-Tumor Effect Mediated by CircRNAs

circRNAs have great potential research value in the occurrence, development, diagnosis, prognosis, and treatment of tumors. Recent studies have revealed that phytochemicals such as curcumin could further exert anti-tumor effects by regulating circRNAs that are engaged in biological processes, including tumor cell proliferation, apoptosis, migration, invasion, autophagy, chemosensitivity, and radiosensitivity [129]. Considering the pivotal roles of circRNAs combined with drugs in cancer, we found that curcumin could regulate the occurrence and development of various tumors through circRNAs acting on different signaling pathways. The research results of the role of circRNAs in various tumors under the action of curcumin in the past 12 years are summarized in Table 3.

There is a study that indicated that curcumin targets the expression of ITGB1 by downregulating circ-203 PRKCA and adsorbing miR-384, thereby inhibiting the occurrence and development of non-small cell lung cancer (NSCLC) [130]. Zhao et al. have proved that curcumin analog GL63 can inactive the JAK2/STAT3 signaling pathway by mediating the circ-ZNF83/miR-324-5p/CDK16 axis, thereby inhibiting the development of hepatic cellular cancer (HCC) [131]. In addition, a recent study found that curcumin could inhibit the proliferation and promote the apoptosis of ovarian cancer cells by regulating the circ-PLEKHM3/miR-320a/SMG1 axis [132]. This offers a better explanation for the mechanism of circRNAs regulating the occurrence and development of cancer to a large extent as well as provides great insight for us to explore the role of curcumin based on circRNAs for future research.

Meanwhile, we found evidence for the potential role of some circRNAs as diagnostic markers for colorectal cancer, as shown in Table 4. In vitro and in vivo experiments showed that overexpression of the tumor suppressor circ-RHOBTB3 could significantly inhibit the invasion and metastasis of colorectal cancer cells by binding to the HuR (ELAVL1) protein and downregulating its expression, thereby reducing the expression of the downstream target gene PTBP1 [136]. Circ-3823 [137], circ-IL4R [138], and circ-N4BP2L2 [139] were significantly overexpressed in colorectal cancer cells, which indicates a poor prognosis in colorectal cancer patients. They act as a tumor-promoting gene by competitively binding miRNAs to eliminate the inhibitory effect of miRNAs on downstream target genes regulating the proliferation, migration, invasion, and apoptosis of colorectal cancer cells. These circRNAs may become new diagnostic markers or potential treatment targets for colorectal cancer.

Throughout the last decade of research, we have identified a series of circRNAs [142] as biomarkers of oncogenic/tumor-suppressive genes that exert anti-colorectal-cancer functions. Although the regulation of these functional molecules of circRNAs has gradually entered public view, the research on circRNAs has just begun. These circRNAs may provide new evidence for future research on the mechanism of colorectal cancer occurrence and development. Whether those circRNAs are responsible for the treatment of colorectal cancer and how circRNAs regulate the biological effects of tumor cells after the curcumin intervention have still not been studied. There is still plenty of room to explore the effect of curcumin on colorectal cancer through circRNAs.

## 3. Discussion

**Figure 2:**
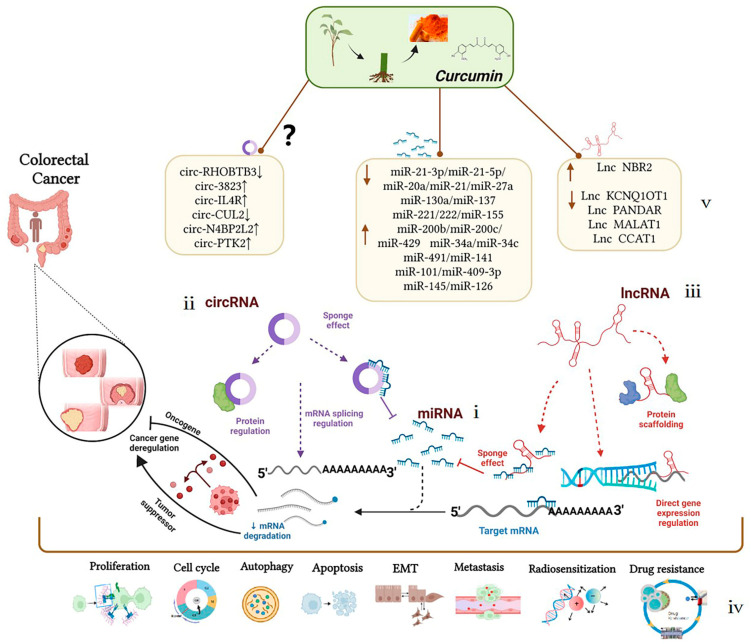
Mechanism of curcumin-targeted regulation of non-coding RNAs against colorectal cancer. Dotted lines represent different regulation methods; boxes represent different ncRNAs regulated by curcumin.

Curcumin can interact with multiple molecular targets through a variety of complex molecular mechanisms to inhibit the growth of tumor cells and achieve anti-colorectal-cancer and chemotherapy sensitization effects. Compared with traditional chemotherapy drugs, curcumin has higher safety levels and is widely used as an ingredient in dietary formulations for the prevention of colorectal cancer. Related clinical trials have been launched. The evidence listed above identifies the mechanism of ncRNAs as potential targets of curcumin for colorectal cancer treatment, summarized in Figure 2. As an explicit regulatory mechanism, miRNAs regulate target mRNAs through complete or incomplete base pairing with 3′UTRs (Figure 2-i), lncRNAs interact with DNAs, RNAs, and proteins to regulate transcription and post-transcriptional processes (Figure 2-ii); circRNAs interact with proteins to regulate the alternative splicing and transcription of target genes (Figure 2-iii). In particular, current studies on ncRNAs regulated by curcumin have been mainly focused on the ceRNA mechanism, also known as the molecular sponge effect, which mainly involves the lncRNAs/circRNAs–miRNAs–mRNAs–proteins pathway. Briefly, target genes can be silenced by miRNA binding. However, ceRNAs, including lncRNAs and circRNAs, can regulate target gene expression by competitively absorbing miRNAs. The mutual regulation between these transcripts (mRNAs and ncRNAs) plays an important role in the occurrence and development of colorectal cancer and mediates biological processes, including colorectal cancer cell proliferation, apoptosis, metastasis, and chemoresistance (Figure 2-iv). As a new research tool or idea, ceRNAs have also been crossed, penetrated, and merged with research in drug-related fields, such as in research on curcumin. We found that curcumin has shown a significant anti-tumor effect in the epigenetic regulation of ncRNAs according to the research progress in the past 12 years. Curcumin could affect the development of colorectal cancer by targeting oncogenes such as miR-130a/miR-137, miR-20a/miR-27a, miR-21, and miR-221/222 or tumor-suppressor genes such as miR-101/miR-409-3p, miR-200b/miR-200c, and miR-34a/miR-34c; its anti-colorectal cancer effect is essentially through the indirect regulation of target genes or signaling pathways. Treated by curcumin, Lnc NBR2, Lnc KCNQ1OT1, Lnc PANDAR, and Lnc CCAT1 could prove to be potentially effective target molecules in the treatment progress of colorectal cancer. Whether a large number of differentially expressed circRNAs, such as circ-3823, circ-IL4R, and circ-CUL2 in colorectal cancer, could become effective targets for curcumin in the treatment of colorectal cancer remains to be further clarified (Figure 2-v). In summary, these findings could provide favorable evidence for exploring the role of curcumin in the treatment of colorectal cancer via non-coding RNAs, which may provide new directions for the treatment and prognosis of colorectal cancer patients. Non-coding RNAs can be potential therapeutic targets for the occurrence and development of colorectal cancer, and curcumin-targeted non-coding RNAs have good biomarker and reference significance for the treatment of colorectal cancer.

However, the efficacy, reliability, and sensitivity of ncRNAs as biomarkers and therapeutic targets for colorectal cancer need further basic research and clinical application. Although different types of ncRNAs have been identified to be involved in the curcumin treatment of colorectal cancer, the existing research has the following issues: (I) The regulation relationship between ncRNAs and curcumin can be found via gene knockdown and overexpression; however, whether this kind of relationship, which exists in a single cell, can be realized in the complex system of the human body still needs to be verified via further clinical trials. (II) Whether curcumin can inhibit the occurrence and development of colorectal cancer through the targeted and precise regulation of the copy number, subcellular localization, and protein binding ability of non-coding RNAs is worthy of further exploration. Additionally, the low stability, low oral bioavailability, and dose-dependent pharmacological effects of curcumin limit its clinical application in cancer therapy and industrialization [143]. Nevertheless, curcumin is a potential candidate compound for anti-tumor drugs due to its clear biological activity and relatively simple molecular structure. The development of a new and more efficient drug delivery system of curcumin will guide its significance in the research on targeting ncRNAs and provide a new prospect for human cancer treatment [144,145,146]. Therefore, for better detection and effective cancer treatment, molecular diagnostic methods combined with drug treatments such as curcumin need to be researched in-depth to enrich their significance and contribute to their clinical application.

Undeniably, more differentially expressed miRNAs, lncRNAs, and circRNAs associated with colorectal cancer need to be further explored. We look forward to more studies on curcumin regulating ncRNAs in colorectal cancer.

## Figures and Tables

**Table 1:** Curcumin modulates miRNAs in colorectal cancer.

In Vitro/ In Vivo	Cell Line	Modulated by Curcumin	Target Gene	Relevant Mechanism	Biological Effects After Administration	Refs.
In vitro/ in vivo	Rko, HCT116, HT-29, SW620	miR-21 ↓ miR-21-3p, miR-21-5p ↓	PDCD4 PTEN ATG10, APAF1	Suppress AP-1 binding to the promoter, p-Akt	Inhibits tumor growth, migration, invasion, metastasis. Promotes autophagy, apoptosis	[96,97,98]
In vitro	SW480	miR-130a ↓	—	Wnt/β-catenin pathway	Inhibits proliferation	[99]
In vitro/ in vivo	HCT116, SW480, HCT116p53^−/−^	miR-34a ↑ miR-27a ↓	CDK4, CDK6, cMyc, FBXW7, Cyclin D1	Deregulation of miRNAs —	Inhibits proliferation, tumor growth, chemoresistant. Promotes cell cycle arrest, apoptosis	[100]
In vitro	HCT-116	miR-491 ↑	PEG10	Wnt/β-catenin pathway	Inhibits proliferation and promotes apoptosis	[101]
In vitro	HCT116−5FUR, SW480-5FUR	miR-141, miR-101, miR-200b, miR-429, miR-200c ↑	— ZEB1 BMI1	—	Inhibits EMT	[102]
In vitro	HCT-116, L-OHP	miR-409-3p ↑	ERCC1	—	Inhibits migration and invasion; promotes apoptosis.	[103]
In vitro	RKO, SW480	miR-20a, miR-27a, miR-17 ↓	ZBTB4, ZBTB10, Sp1, Sp3, Sp4,	—	Inhibits proliferation	[104]
In vitro/in vivo	SW620, HCT116, HCT116wt, HCT116 p53^−/−^	miR-34a ↑ miR-34c ↑	Notch-1	—	Inhibits proliferation, promotes apoptosis	[105]
In vitro/in vivo	SW480	miR-145 ↑ (Nano-CUR)	—	—	Interferes with tumor growth, inhibits proliferation, migration	[106]
In vitro/in vivo	HCT116, LoVo, HT29-MTX	miR-31 (PS-TP-miR-31i/Cur NPs)	—	—	Inhibits cell proliferation, tumor growth	[107]
In vitro	HT-29, HCT-116, LoVo, SW480, DLD-1 CRL-1790	miR-137 ↓	GLS	GLS–gluamine Metabolism	Increases cell death, anti-chemoresistance	[108]
In vitro	HT-29	miR-21, miR-155, miR221/222 ↓ miR-34a, miR-126 ↑	—	—	Promotes apoptosis	[109]

Note: Arrows “↓, ↑” represent the expression levels of miRNAs regulated by curcumin in CRC. “↑”: upregulated, “↓”: downregulated.

**Table 2:** Curcumin modulates lncRNAs in colorectal cancer.

In Vitro/ In Vivo	Cell Line	Modulated by Curcumin	Relevant Mechanism	Biological Effects after Administration	Refs.
In vitro	HCT116, SW480	NBR2 ↑	Activates of AMPK pathway	Inhibits proliferation	[119]
In vitro/ In vivo	HCT8 DDP cells	KCNQ1OT1 ↓	Sponge of miR-497 increases Bcl-2 expression	Inhibits proliferation, promotes apoptosis, chemoresistant	[122]
In vitro	DLD-1, SW620, HCT116	PANDAR ↓	PUMA upregulation	Promotes apoptosis	[125]
In vitro/ In vivo	DLD-1	PANDAR ↓	Induces PUMA	Promotes apoptosis, reduces cell aging	[126]
In vitro	SW480	MALAT1 ↓ (Si-MALAT1)	Downregulated c-myc, cyclinD1, β-catenin	Inhibits cell viability, migration, invasion	[127]
In vitro/ In vivo	HT-29	CCAT1 ↓ (Si-CCAT1-CSNP)	—	Inhibits proliferation, migration, induces apoptosis	[128]

Note: Arrows “↓, ↑” represent the expression levels of lncRNAs regulated by curcumin in CRC. “↑”: upregulated, “↓”: downregulated.

**Table 3:** Curcumin modulates circRNAs in various cancers.

In Vitro/ In Vivo	Cell Line	Modulated by Curcumin	Relevant Mechanism	Biological Effects after Administration	Refs.
In vitro/ In vivo	H1650, H1299, H460, A549, 16HBE (NSCLC)	circ-PRKCA ↓	circ-PRKCA/miR-384/ITGB1 pathway	Inhibits viability, colony formation, migration, invasion, promotes apoptosis	[130]
In vitro/ In vivo	THLE-2, HuH-7, HCCLM3 (HCC)	circ-ZNF83 ↓	JAK2/STAT3 pathway circZNF83/miR-324-5p/CDK16 axis	Inhibits proliferation, cell cycle, migration, invasion, promotes apoptosis	[131]
In vitro/ In vivo	SKOV3, A2780, IOSE-80 (Ovarian cancer)	circ-PLEKHM3 ↑	circ-PLEKHM3/miR-320a/SMG1 axis	Inhibits proliferation and promotes apoptosis	[132]
In vitro/ In vivo	CAKI-1, ACHN, (RCC)	circ-FNDC3B ↓	circ-FNDC3B/miR-138-5p/IGF2 axis	Inhibits proliferation, promotes apoptosis	[133]
In vitro	CNE-2 (NPC)	circRNA-102115	circRNA-102115/miR-335-3p/MAPK1	Enhances radiosensitization	[113]
In vitro	CNE-2 (NPC)	circRNA network	miRNA sponge regulated EGFR, STAT3, GRB2	Enhances radiosensitization	[134] [135]

Note: Arrows “↓, ↑” represent the expression levels of circRNAs regulated by curcumin in various cancers. “↑”: upregulated, “↓”: downregulated.

**Table 4:** Roles of circRNAs in colorectal cancer.

In Vitro/In Vivo	Cell Line	circRNAs in CRC	Target Gene	Relevant Mechanism	Biological Effects	Refs.
In vitro/ In vivo	RKO, HCT116, SW480, SW620, DLD-1, HT29, Colo320, HCE8693	circ-RHOBTB3 ↓	PTBP1, FUS ADARB2	circ-RHOBTB3/HuR/PTBP1 protein ubiquitination	Restrains metastasis, invasion	[136]
In vitro/ In vivo	HCT116, SW480	circ-3823 ↑	TCF7	circ-3823/miR-30c-5p/TCF7 miRNA sponge m6A modification	Promotes proliferation, metastasis, angiogenesis	[137]
In vitro	FHC, HCT116, DLD1, LoVo, SW620, HT29, SW480	circ-IL4R ↑	PHLPP1	circ-IL4R/PI3K/AKT, miRNA sponge, protein ubiquitination	Promotes proliferation, migration, invasion	[138]
In vitro/ In vivo	HT-29, SW480, HCT-116, LoVo, NCM460	circ-N4BP2L2 ↑	CXCR4	circ-N4BP2L2/miR-340-5p/ CXCR4 (miRNA sponge)	Promotes proliferation, migration, invasion. Promotes tumor growth, metastasis	[139]
In vitro/ In vivo	HT290, HCT116, SW480, SW620, FHC	circ-CUL2 ↓	PPP6C	circ-CUL2/miR-208a-3p/PPP6C (miRNA sponge)	Inhibits proliferation ability, induces apoptosis, autophagy	[140]
In vitro/ In vivo	HT-29, LoVo, SW480, HCT-116, NCM460	circ-PTK2 ↑	YTHDF1	circ-PTK2/miR-136-5p/YTHDF1	Promotes proliferation, migration, invasion, chemoresistance	[141]

Note: Arrows “↓, ↑” represent the expression levels of circRNAs in CRC. “↑”: upregulated, “↓”: downregulated.

## Data Availability

Not applicable.
